# Trends and determinants of antenatal, intranatal, and postnatal care utilization among Indonesian women (2012–2017): associated factors in midwifery service utilization between midwives and other health professionals

**DOI:** 10.1186/s12912-025-03527-6

**Published:** 2025-07-14

**Authors:** Qorinah Estiningtyas Sakilah Adnani, Rufidah Maulina, Kadar Ramadhan, Niken Bayu Argaheni, Holly Powell Kennedy, Michelle Telfer

**Affiliations:** 1 Department of Public Health, Faculty of Medicine, Padjadjaran University, Bandung, Indonesia; 2 Midwifery Study Program, Faculty of Medicine, Universitas Sebelas Maret, Surakarta, Indonesia; 3 Department of Midwifery, Poltekkes Kemenkes Palu, Palu, Indonesia; 4 School of Nursing, Yale University, New Haven, Connecticut, 06477 USA

**Keywords:** Midwives, Maternal health, Antenatal care, Intranatal care, Postnatal care, Maternal mortality, Indonesia

## Abstract

**Background:**

Maternal healthcare services during pregnancy and childbirth are essential to reducing adverse maternal and child health outcomes, including maternal and child mortality. Midwives in Indonesia play an important role in providing maternal healthcare services.

**Purpose:**

The purpose of this study is to analyze trends in midwifery care utilization for antenatal care (ANC), intranatal care (INC), and postnatal care (PNC) among Indonesian women between in 2012 and 2017, and to examine the sociodemographic, economic, and regional factors associated with access and utilization to inform strategies for improving maternal and neonatal health outcomes.

**Method:**

This study uses data from the Indonesia Demographic and Health Survey (IDHS) from 2012 to 2017. A total of 15,269 women aged 15–49 from the Indonesia Demographic and Health Survey (IDHS) participated in this study, consisting of 7,712 women from the 2012 survey and 7,557 from the 2017 survey. Only respondents who completed the Women’s Questionnaire were included in the analysis. A multiple logistic regression model was used to analyze factors associated with midwives’ engagement in maternal healthcare service provision. This study used multivariate analyses for 2012 and 2017 to examine the relationships between various socio-demographic characteristics and the utilization of specific maternal health services in Indonesia.

**Result:**

Several factors consistently influenced service utilization of midwifery services, including younger maternal age, secondary education, being married, without health insurance, rural residence, and higher ANC visit frequency. Women with normal births and without complications were significantly utilizing the service from midwives. Conversely, disparities remained for women in the eastern part of Indonesia and among those with higher parity.

**Conclusion:**

Midwives play a critical role as accessible and trusted providers and remain central to strengthening service delivery and extending community-level reach across Indonesia. These insights offer valuable guidance for policymakers and health planners in enhancing midwife-led service coverage and improving maternal health outcomes.

**Clinical trial number:**

Not applicable.

## Introduction

Midwives are essential in preventing maternal mortality in Indonesia by managing normal births and managing and referring those with birth complications. In Indonesia, midwives (*bidan*) are recognized as qualified health professionals skilled in overseeing normal pregnancies, childbirth, and the immediate postnatal phase, as well as in identifying complications [1]. Conversely, traditional birth attendants (*dukun*) generally acquire their abilities via apprenticeship instead of formal education and may lack equivalent expertise in midwifery care [2, 3]. Because of this concern, the Indonesian government has progressively acknowledged midwives above traditional birth attendants in its efforts to mitigate the persistently high maternal death rates in the country [4]. Then, multiple measures were adopted to enhance access to trained birth attendants, particularly midwives, in response to these problems.

The strategy started in the 1990s, when the Indonesian government established a program to educate midwives, especially in remote regions with restricted healthcare access [4]. This approach is integral to a comprehensive strategy aimed at guaranteeing that all births are accompanied by skilled birth attendants, therefore improving maternal and newborn health outcomes and mitigating the issues associated with unlicensed birth attendants [4]. For example, efforts to reduce maternal mortality by improving reproductive health services, with antenatal care (ANC) as a key preventive measure, and ensuring births are attended by skilled birth attendants (SBAs) [5]. The availability and accessibility of SBAs have the potential to save mothers from pregnancy-related complications [6]. The presence of SBAs during labour and birth, and postpartum, with the expertise necessary to manage care for both mothers and newborns [7]. While these programs provide a foundation for improving maternal healthcare, the role of midwives within this system is vital in delivering comprehensive care.

Midwives’, whether they work in a remote village, a private clinic, a primary care clinic, or a hospital, have a scope of practice that includes providing timely care and management for normal and abnormal antenatal, intrapartum, and postpartum care [8]. To enhance these services further, recent changes in national healthcare guidelines have redefined antenatal care practices in Indonesia. In Indonesia, the healthcare system, dating back to 1960, has involved sub-district health centers (*Puskesmas*) and village health posts (*Pustu*) in providing primary maternal healthcare services, both in urban and rural areas [6, 9]. Furthermore, the Village Midwife program established by the Indonesian government in 1989 aimed to ensure that every village has access to maternal health services before, during, and after childbirth, with the assistance of trained midwives [10]. This initiative aimed to reduce the incidence of maternal mortality and improve indicators of maternal health coverage, such as births assisted by health professionals, antenatal visits, and postpartum care [11], which has resulted in an increasing number of prenatal care visits and improved the health status of women [12].

Despite these improvements, barriers to accessing maternal healthcare remain, particularly for postnatal care. Access to available postnatal health services is hindered due to regional inequities, cultural beliefs in not going outside before 40 days postpartum, women justifying a healthy baby, women being overconfident about the baby’s condition, and experienced family members who take care of their baby [13, 14]. These factors contribute to missed opportunities for critical health interventions during the postpartum period. Addressing these barriers is essential for improving maternal and child health outcomes, which remain a global priority.

Enhancing maternal and neonatal health while decreasing mortality rates is an essential global concern. Maternal health profoundly influences outcomes for both mothers and infants, as evidenced by the World Health Organization’s (WHO) study, which indicated a maternal mortality ratio (MMR) of 211 deaths per 100,000 live births in 2017 [15]. Indonesia experienced a significant reduction in its maternal mortality ratio (MMR) over the past decade. According to the 2012 IDHS, the MMR was 359 deaths per 100,000 live births [16]. Statistics Indonesia later reported a decline to 305 deaths per 100,000 live births based on the 2015 SUPAS [17], followed by a further decrease to 189 deaths per 100,000 live births in the 2020 Long Form Population Census [18]. The ongoing need for initiatives to improve maternal health is underscored by the fluctuation of MMR in Indonesia, which is predominantly caused by pregnancy, childbirth, and postpartum-related issues [19]. One of the strategies to reduce maternal mortality is closely linked to the role of skilled birth attendants, particularly midwives, in providing ANC, INC, and PNC.

Community-based midwifery services can mitigate maternal mortality by implementing a continuity of care framework. Skilled birth attendants, particularly midwives, are able to monitor maternal health, identify potential complications early, and educate women about safe delivery practices through regular check-ups. Continuity models allow for trust to be built between the midwife and the woman, which then allows for personalised care and empowerment of the woman. This relationship is essential to improved clinical outcomes [20]. Additionally, midwives in health facilities provide immediate care to women during labor and childbirth, a critical phase during which the majority of maternal deaths occur [21]. After childbirth, midwives provide postpartum care to monitor and manage any complications that may arise, thereby assuring the health of both the mother and the newborn. In low- and middle-income countries (LMICs), maternal and newborn mortality could be reduced by 30–80% with adequate coverage of midwifery interventions, according to a Lancet series on midwifery [22]. As a result of the training of midwives in Guatemala, maternal mortality decreased by 27% in less than a decade [23].

The use of maternal healthcare services—including antenatal care (ANC), intranatal care (INC), and postnatal care (PNC)—is shaped by a range of factors at the individual, household, and broader systemic levels. To understand these influences, this study draws on Andersen’s Behavioral Model of Health Services Use, which offers a well-established framework for examining health service utilization [24]. According to this model, three main categories of factors influence whether individuals seek care: predisposing factors (such as age, education, and parity), enabling factors (including economic status, health insurance, and access to information), and need-related factors (such as perceived health conditions or complications) [25]. In this context, the study examines the predisposing characteristics such as women’s age, level of education, marital status, and number of children, along with enabling conditions like place of residence, insurance coverage, and household wealth, contribute to differences in the use of midwifery services by midwife across ANC, INC, and PNC.

In Indonesia, most childbirths are assisted by midwives in health care facilities. The results of the 2017 IDHS show that 91% of live births were assisted by trained healthcare professionals. Midwives provided assistance for 61% of these births, followed by obstetricians at 29%, and general practitioners at 1% [26]. This highlights midwives’ critical role in reducing maternal and neonatal mortality and ensuring safe births. Evidence from LMICs also demonstrates that midwives contribute significantly to reductions in maternal and neonatal mortality and stillbirths [22]. This contribution may have influenced the MMR reduction in Indonesia between 2012 and 2017, although further evidence is needed. However, the effectiveness of midwifery services also depends on the extent to which they are utilized across different stages of care. Service utilization rates continue to be suboptimal, despite these advancements [27]. Approximately 77% of Indonesian mothers underwent at least four ANC visits in 2017, which was marginally higher than the government’s objective of 76% [28]. This represented an increase from 74% in 2012. Despite the government’s objective of achieving 79% utilization of INC services, it only managed to achieve 74% in 2017. Nevertheless, this constituted an improvement from the 63% in 2012. In 2012, the utilization of PNC services was 80%, and it increased marginally to 87% in 2017 [26, 28, 29].

Although numerous studies have explored maternal healthcare utilization in Indonesia and other low- and middle-income countries, most have focused on individual components of care—such as antenatal care (ANC) or skilled birth attendance—without assessing the continuum of care that includes intranatal (INC) and postnatal care (PNC) [30, 31]. Furthermore, many analyses aggregate healthcare providers into a single category of “skilled birth attendants,” which overlooks the unique role midwives play compared to nurses, general practitioners, and obstetricians [30, 32]. This study offers a novel contribution by specifically disaggregating maternal healthcare utilization by type of provider and analyzing midwifery care separately for ANC, INC, and PNC. Using nationally representative data from the 2012 and 2017 Indonesia Demographic and Health Surveys (IDHS), the study applies multivariate logistic regression to examine the sociodemographic, economic, and geographic determinants associated with choosing midwives over other health professionals. By bridging these gaps, this research provides essential evidence to support targeted health policies and interventions that aim to optimize and expand the role of midwives in Indonesia’s maternal healthcare system.

Given the significance of ANC, INC, and PNC conducted by midwives in preventing maternal and newborn deaths, this study aims to investigate the factors associated with midwives’ involvement in maternal health services for women of childbearing age in Indonesia from 2012 to 2017.

## Method

### Data source

The study utilized data from the Indonesia Demographic and Health Surveys (IDHS), which is a nationally conducted cross-sectional survey every five years, about the demographic and health status of the Indonesian population. IDHS is overseen by Statistics Indonesia, the Ministry of Population and Family Development/ BKKBN (Badan Kependudukan dan Keluarga Berencana), and the Ministry of Health. Sampling Methods: The study employed a two-stage stratified random sampling method to select a nationally representative sample of households. This means that the researchers did not include all households but selected a subset in a systematic way. First Stage of Sampling: In the first stage of sampling, the entire population (households) was divided into strata or groups. This stratification helps ensure that different segments of the population are represented in the sample. Second Stage of Sampling: Within each stratum, households were selected using random sampling methods. Inclusion Criteria: Women who met the eligibility criteria from the selected households were included in the study.

### Analytical sample

This study examined data from female participants aged 15 to 49 who took part in the Indonesia Demographic and Health Survey (IDHS) utilizing the Women’s Questionnaire (Individual Record). The 2012 survey had 7,712 women out of 8,490 respondents. In the 2017 study, 7,557 women were examined out of 10,239 respondents. The unit of analysis was women aged 15–49 with their last child in the past five years. Inclusion criteria for this study were: [1] women aged 15–49 years; [2] had at least one live birth in the five years preceding the survey; [3] only the most recent (last) birth was analyzed to minimize recall bias; [4] received antenatal care (ANC) visits for the most recent pregnancy; [5] received antenatal care (ANC), intrapartum care (INC) and postnatal care (PNC) services from a skilled provider, with provider type specified (midwife, nurse, doctor, and obstetrician); [6] gave birth to a singleton live baby; and [7] delivery occurred in a health facility or was attended by a skilled health provider. Respondents were eliminated if they did not complete the questionnaire, had incomplete data, or selected “do not know” as a response during the final data-cleaning procedure.

### Variables

The independent variables in this study were individual characteristics such as age, age at first childbirth, educational attainment, employment status, parity, ownership of insurance, and the wealth index [33]. Additionally, we examined the spouse’s education level, marital status, access to mass media (comprising reading newspapers or magazines, watching television, and listening to the radio), and the classification of residence into urban or rural areas, and which part of Indonesia the respondents live in. For this study, we compare the analysis by midwives’ assisted care and other health professionals’ care in order to understand the factors differentiating midwives from other health personnel in 2012 and 2017.

The dependent variable was midwifery care, which is ANC. INC and PNC (provide a more straightforward explanation about the dependent variable: Y = 1 if utilizing midwifery services for ANC, INC, and PNC, and Y = 0 if utilizing others. Others were medical doctors, obstetricians, and nurses who were helping women, defined below:

1. Definition and Importance:

**Antenatal Care (ANC)**:

#### Definition

Antenatal Care (ANC) refers to the healthcare services provided to pregnant women before childbirth. It encompasses a series of health visits, including screenings and interventions that aim to improve the health of the expectant mother, ensure the well-being of the developing fetus, and identify and manage any potential complications that may arise during pregnancy. The indicator was the women who had a live birth in the past five years and received antenatal care from a midwife and another health professional, including nurses, doctors, and obstetricians. Therefore, the indicator for ANC was divided into two.

0 = other health professionals (nurse, doctor, and obstetrician).

1 = Midwife.

**Intranatal Care (INC)**:

#### Definition

Intranatal Care (INC) involves the care and support provided to pregnant women during childbirth, from the onset of labor to the birth of the baby. In this study, the data included was INC, which was provided by a midwife and another health professional, including nurses, doctors, and obstetricians, during the last five years. Therefore, the indicator for INC was divided into two.

0 = other health professionals (nurse, doctor, and obstetrician).

1 = Midwife.

**Postnatal Care (PNC)**:

#### Definition

Postnatal Care (PNC) comprises the healthcare services and support provided to both mothers and newborns in the six weeks following childbirth. It focuses on monitoring the physical and emotional well-being of the mother, addressing postpartum complications, and ensuring the healthy development of the newborn. PNC was categorized based on the timing of the initial postnatal examination by a healthcare provider for the mother (less than 4 h, 4–23 h, 1–2 days, 3–6 days, 7–41 days) and whether she received a postnatal check during the first 2 days following childbirth. Postnatal Care was provided by a midwife and another health professional, including nurses, doctors, and obstetricians, during the last two years. Therefore, the indicator for PNC was divided into two.

0 = other health professionals (nurse, doctor, and obstetrician).

1 = Midwife.

Operational Definition of Independent Variables:

- Maternal Age: Refers to the current age of women aged 15–49 years at the time of the survey.
- Age at First Birth: Indicates the age at which a woman gave birth to her first child.
- Level of Education: Denotes the highest level of formal education attended or completed by women aged 15–49, categorized as no education, primary, secondary, or higher, with corresponding data on median years of schooling.
- Employment Status: Refers to women who were working in the seven days prior to the survey, or who were not working in the past week but had been employed in the past seven months.
- Husband’s Level of Education: Refers to the highest level of formal education attained by the woman’s husband or partner, categorized similarly to the respondent’s education level.
- Marital Status: Represents women who were legally married or living with a partner as if married at the time of the survey.
- Parity: Indicates the total number of live births a woman has had during her lifetime.
- Health Insurance: Identifies whether the respondent is covered by health insurance, either through government-sponsored schemes such as JKN/BPJS (subsidized or non-subsidized) and Jamkesda, or through private insurance providers.
- Complication: Refers to any health problems or complications experienced by women aged 15–49 during their most recent live birth within the five years preceding the survey.
- ANC Visit (Antenatal Care): Represents the number of visits received during pregnancy from skilled health providers, including doctors, midwives, or nurses, which are no visit, less than 4 times, and equal to or more than 4 times.
- Birth Delivery Type: Categorized into vaginal (normal) birth delivery and cesarean section (sectio), based on the method by which the baby was delivered.
- Mass Media Exposure– Newspapers or Magazines: Women who read newspapers or magazines at least once a week are considered regularly exposed to print media.
- Radio Listening: Women who listen to the radio at least once a week are categorized as having regular exposure to radio broadcasts.
- Watching TV: Regular exposure to television is defined as watching TV at least once a week.
- Wealth Index: A composite measure of a household’s living standard, calculated using principal component analysis based on asset ownership (e.g., television, bicycle), housing conditions (e.g., flooring, toilet facilities), and access to water. The index is divided into quintiles from the lowest to the highest.
- Type of Residence: Classifies respondents based on whether they live in urban or rural areas.
- Province: Geographical classification of respondents’ residence into major regions of Indonesia: western, central, and eastern provinces.

### Statistical analysis

An univariate analysis was performed to delineate the proportion and frequency of each variable, specifically demographic variables and skilled birth attendants (midwives and other healthcare professionals). Bivariate analyses were used to investigate demographic and socioeconomic status among the research participants. A multivariate analysis was performed using multiple logistic regression models to identify an association between multiple independent variables, both categorical and continuous, simultaneously [34]. Odds ratios (ORs) and their respective 95% confidence intervals (CIs) were calculated using multiple logistic regression. Statistical significance was established at a *p*-value threshold of less than 0.05. Statistical analysis was performed using Stata 17.0 software.

## Results

This study examines trends in the utilization of midwifery services for antenatal care (ANC), intranatal care (INC), and postnatal care (PNC) among Indonesian women from 2012 to 2017. The objective is to delineate the sample characteristics by univariate analysis and investigate the sociodemographic, economic, and regional aspects affecting access and utilization, thereby informing initiatives to enhance maternal and neonatal health outcomes.

Table 1 presents detailed results of the bivariate association between the socioeconomic, demographic, and obstetric factors between ANC, INC, and PNC during 2012. In 2012, 56.6% of Indonesian women received antenatal care from a midwife, while 69.3% were attended by a midwife during childbirth, and 70.4% accessed postnatal care services provided by a midwife. Women aged 15 to 19 exhibited the greatest utilization of midwifery services from a midwife for ANC at 58.5%, INC at 68.2%, and PNC at 77.7%, with a gradual decrease as age advanced. Women who experienced their first delivery between the ages of 10 and 19 demonstrated greater utilization of midwifery services for ANC (57.9%), INC (66.0%), and PNC (74.4%) than those whose first birth took place at older ages. Education significantly influenced midwife utilization, with women possessing primary education exhibiting greater rates for ANC (56.8%), INC (63.3%), and PNC (74.5%), whereas those lacking education showed markedly lower utilization rates, especially during INC (27.6%). The employment status revealed a minor discrepancy, as non-working women accessed midwifery services from a midwife for PNC (73.5%) more frequently than working women (67.7%).

Additional characteristics affecting service utilization included the husband’s educational attainment, with women married to husbands with secondary education showing greater use of midwifery care from midwives for INC (75.2%) and PNC (73.1%) compared to husbands with no formal education.

Rural women utilized midwifery services from midwives for ANC (69.2%) and PNC (75.6%) more than their urban counterparts. Regional discrepancies were apparent, with provinces in the western and central regions of Indonesia exhibiting greater utilization of midwifery services, especially for ANC (63.6% in the west), whereas the eastern area demonstrated markedly lower usage (10.2%). The wealth index indicated that economically disadvantaged women exhibited less midwifery utilization from midwives for ANC and PNC in comparison to their richer counterparts, underscoring socio-economic disparities.

Table 1 reveals substantial variations in the utilization of midwifery services influenced by demographic and socioeconomic characteristics. Women who are younger, possess higher education, belong to affluent households, and have husbands with advanced education are more inclined to utilize midwifery services. Conversely, older women, those with lower educational attainment, and individuals from economically disadvantaged households experience reduced access, underscoring disparities and obstacles in maternal healthcare.

Prevalence: While pregnancies outside of marriage are not legally permitted, it is important to note that they do occur in Indonesia, as they do in every society around the world. The prevalence of such pregnancies can vary depending on cultural, social, and economic factors. There may be instances where couples choose to have children without formalizing their marital status or where pregnancies occur unintentionally or as a result of assault or rape before marriage. It’s worth mentioning that societal views on this issue can also vary, and some communities or individuals may be more accepting or understanding of such situations than others. Sexual assault and rape also play a role in pregnancy outside of marriage and are likely underreported depending on legal, cultural and religious norms. Overall, while pregnancies outside of marriage are not legally permitted in Indonesia, they still happen, and the societal response to such situations may differ among individuals and communities.

**Table 1:** Percentage distribution of ANC, INC, and PNC services by background characteristics: Indonesia DHS 2012 (*n* = 7712)

Characteristics	2012														
	ANC					INC					PNC				
	Other		Midwife		*p*-value	Other		Midwife		*p*-value	Other		Midwife		*p*-value
	n (7,712)	% (43.4)	n (6,376)	% (56.6)		n (5,042)	% (30.7)	n (9,659)	% (69.3)		n (3,006)	% (29.6)	n (6,463)	% (70.4)	
**Age**															
15–19	271	41.5	222	58.5	< 0.001	202	31.8	325	68.2	< 0.001	87	22.3	228	77.7	< 0.001
20–24	1400	38.7	1292	61.3		980	29.7	1851	70.3		509	24.2	1293	75.8	
25–29	2055	40.5	1886	59.5		1269	27.6	2790	72.4		786	27.0	1880	73.0	
30–34	1935	45.5	1504	54.5		1230	32.5	2332	67.5		769	33.7	1539	66.3	
35–39	1341	48.4	994	51.6		840	30.5	1600	69.5		557	33.0	1027	67.0	
40–44	595	49.3	404	50.7		423	36.9	644	63.1		251	35.8	418	64.2	
45–49	115	45.6	74	54.4		98	42.8	117	57.2		47	33.8	78	66.2	
**Age at 1st birth**															
10–19	2415	42.1	1983	57.9	< 0.001	1834	34.0	2896	66.0	< 0.001	790	25.6	2040	74.4	< 0.001
20–24	3331	41.0	3016	59.0		1986	26.4	4582	73.6		1191	25.1	3112	74.9	
25–29	1497	48.9	1085	51.1		892	32.3	1727	67.7		765	39.7	1057	60.3	
30–34	378	51.9	243	48.1		263	42.5	374	57.5		202	49.2	214	50.8	
35–49	91	52.8	49	47.2		67	46.2	80	53.8		58	65.4	40	34.6	
**Level of education**															
No education	166	61.7	75	38.3	< 0.001	278	72.4	105	27.6	< 0.001	46	42.3	78	57.7	< 0.001
Primary	2276	43.2	1930	56.8		1913	36.7	2594	63.3		737	25.5	1911	74.5	
Secondary	3921	38.5	3795	61.5		2144	24.4	5743	75.6		1485	26.0	3779	74.0	
Higher	1349	63.7	576	36.3		707	37.5	1217	62.5		738	52.9	695	47.1	
**Employment status**															
Not working	3483	40.7	3049	59.3	< 0.001	2175	28.1	4585	71.9	< 0.001	1257	26.5	2924	73.5	< 0.001
Working	4229	45.9	3327	54.1		2867	33.0	5074	67.0		1749	32.3	3539	67.7	
**Husband’s level of education**															
No education	126	49.7	61	50.3	< 0.001	202	60.5	92	39.5	< 0.001	34	31.1	67	68.9	< 0.001
Primary	2253	41.7	1952	58.3		1825	36.5	2669	63.5		719	24.2	1943	75.8	
Secondary	4050	40.0	3831	60.0		2318	24.8	5763	75.2		1537	26.9	3805	73.1	
higher	1283	63.2	532	36.8		697	38.5	1135	61.5		716	53.2	648	46.8	
**Marital status**															
Married	7359	43.0	6216	57.0	< 0.001	4795	30.5	9330	69.5	0.042	2893	29.6	6255	70.4	0.939
other	353	55.5	160	44.5		247	36.6	329	63.4		113	29.9	208	70.1	
**Parity**															
Primigravida	2508	38.4	2469	61.6	< 0.001	1621	28.2	3477	71.8	< 0.001	1112	28.8	2327	71.2	0.575
Multigravida	4485	45.9	3518	54.1		2780	30.8	5529	69.2		1688	30.2	3674	69.8	
Grand multigravida	719	51.8	389	48.2		641	43.9	653	56.1		206	29.2	462	70.8	
**Health insurance**															
No	4090	38.2	4264	61.8	< 0.001	2996	29.7	5827	70.3	0.021	1494	26.0	3897	74.0	< 0.001
yes	3622	52.3	2112	47.7		2046	32.6	3832	67.4		1512	25.3	2566	74.7	
**Complication**															
Yes	989	42.0	821	58.0	0.355	656	33.2	1192	66.8	0.090	521	38.7	726	61.3	< 0.001
No	6723	43.6	5555	56.4		4386	30.4	8467	69.6		2485	28.2	5737	71.8	
**ANC Visit**															
no visits						483	75.1	138	24.9	< 0.001	83	52.7	89	47.3	< 0.001
< 4 times	1005	47.8	611	52.2	0.014	812	46.6	806	53.4		250	28.3	526	71.7	
≥ 4 times	6707	43.0	5765	57.0		3747	27.8	8715	72.2		2673	29.4	5848	70.6	
**Delivery Birth** ** type**															
Normal	N/A					3921	26.4	8956	73.6	< 0.001	2056	22.6	6100	77.4	< 0.001
Section						1121	60.1	703	39.9		950	73.4	363	26.6	
**Mass Media consumption**															
**News papers or magazines**															
No	3757	41.3	3199	58.7	0.001	2836	32.8	4639	67.2	< 0.001	1229	26.1	3131	73.9	< 0.001
Yes	3955	45.6	3177	54.4		2206	28.4	5020	71.6		1777	32.9	3331	67.1	
**Radio listening**															
No	3884	42.8	3138	57.2	0.417	2747	32.1	4697	67.9	0.023	1353	27.5	3127	72.5	0.006
Yes	3828	43.9	3238	56.1		2295	29.4	4962	70.6		1653	31.4	3336	68.6	
**Watching TV**															
No	446	60.0	183	40.0	< 0.001	492	53.1	320	46.9	< 0.001	94	26.0	222	74.0	0.349
Yes	7266	42.8	6193	57.2		4550	29.8	9339	70.2		2912	29.7	6241	70.3	
**Wealth index**															
Lowest	2441	56.2	1261	43.8	< 0.001	2083	46.6	2076	53.4	< 0.001	661	27.0	1584	73.0	< 0.001
Second	1536	41.8	1372	58.2		914	28.4	2082	71.6		509	23.2	1468	76.8	
Middle	1258	35.1	1408	64.9		648	21.9	2074	78.1		470	22.5	1306	77.5	
Fourth	1172	35.7	1374	64.3		621	23.7	1939	76.3		543	27.6	1251	72.4	
Highest	1305	49.5	961	50.5		776	33.1	1488	66.9		823	46.0	854	54.0	
**Type of residence**															
Urban	3602	44.2	3045	55.8	0.327	1977	28.1	4753	71.9	< 0.001	1691	34.2	2908	65.8	< 0.001
Rural	4110	42.6	3331	57.4		3065	33.3	4906	66.7		1315	24.4	3555	75.6	
**Province**															
Western Indonesia	3412	36.4	5111	63.6	< 0.001	2523	28.4	6209	71.6	< 0.001	1662	28.9	3958	71.1	< 0.001
Central Indonesia	3193	72.9	1141	27.1		1722	37.2	2780	62.8		1051	31.6	2062	68.4	
Eastern Indonesia	1107	89.8	124	10.2		797	62.2	670	37.8		293	43.4	443	56.6	

Table 2. showed bivariate association between socioeconomic, demographic, and obstetric factors between ANC, INC, and PNC during 2017. In 2017, 58% of all Indonesian women received ANC from a midwife, 73.5% delivered INC with a midwife, and 74.1% PNC from a midwife. According to Table 2, younger women (15–19 years) demonstrated the highest utilization rates for midwifery services from midwife, with 60.9% for ANC, 78.7% for INC, and 77.8% for PNC. In contrast, older age groups, particularly those aged 40–44 years, exhibited lower utilization rates: 51.5% for ANC, 70.1% for INC, and 74.3% for PNC. Women who experienced their initial childbirth between the ages of 10 and 19 demonstrated greater utilization of midwifery services from midwife for ANC (58.9%), INC (76.3%), and PNC (79.7%) in comparison to those who gave birth for the first time at later ages. The degree of education significantly influenced the utilization of midwifery services from midwife, with women possessing secondary education accessing ANC at 62.6%, INC at 76.4%, and PNC at 76.9%. In contrast, women with no formal education exhibited markedly lower rates, especially for ANC at 30.6% and INC at 59.2%. The employment status affected utilization midwifery services, with non-working women exhibiting somewhat higher rates for ANC (60.2%) and PNC (76.5%) than their working counterparts (55.9% for ANC and 72.0% for PNC).

Socioeconomic and geographic disparities were also evident. Uninsured women demonstrated higher utilization of midwifery services from midwife for ANC at 66.0% and PNC at 79.2%, in contrast to women who have insurance who utilized these services at rates of 52.6% for ANC and 70.7% for PNC. Media exposure demonstrated a beneficial impact, as women who viewed television accessed midwifery services from midwife for ANC (58.6%) and PNC (74.1%) at higher rates than those who did not engage with television. Regional disparities were significant, as western provinces exhibited a higher utilization of midwifery services for ANC (65.4%) in contrast to eastern provinces, which recorded the lowest rates (8.3%). The wealth index further underscored disparities, as richer groups exhibited greater utilization of all services, with ANC rates at 66.0% for the middle wealth quintile, in contrast to 46.7% for the lowest quintile. These data highlight the impact of demographic, socioeconomic, and regional determinants on the utilization of maternal healthcare in Indonesia.

The results of bivariate analysis revealed significant relationships between the utilization of health services provided by midwives and various determinants among women in the reproductive age group in Indonesia. Unsurprisingly, women aged 19–39 are more likely to use health services with the assistance of midwives compared to women over 39 years old. Moreover, women giving birth for the first time between the ages of 20 and 34, those with higher education levels, and those who are employed were more likely to engage with midwives for health services.

**Table 2:** Percentage distribution of ANC, INC, and PNC services by background characteristics: Indonesia DHS 2017 (*n* = 7557)

Characteristics	2017														
	ANC					INC					PNC				
	Other		Midwife		*p*-value	Other		Midwife		*p*-value	Other		Midwife		*p*-value
	n (7557)	% (42.0)	n (6640)	% (58.0)		n (3,719)	% (26.5)	n (10,550)	% (73.5)		n (2,474)	% (25.9)	n (7,228)	% (74.1)	
**Age**															
15–19	179	39.1	161	60.9	< 0.001	69	21.3	271	78.7	< 0.001	41	22.2	165	77.8	0.108
20–24	1085	35.9	1124	64.1		509	22.4	1703	77.6		336	22.4	1164	77.6	
25–29	1837	40.2	1741	59.8		896	25.2	2703	74.8		662	27.3	1802	72.7	
30–34	2032	43.6	1706	56.4		990	27.1	2767	72.9		646	25.8	1950	74.2	
35–39	1545	43.7	1294	56.3		814	29.6	2041	70.4		536	27.5	1385	72.5	
40–44	723	48.5	514	51.5		364	29.9	882	70.1		207	25.7	629	74.3	
45–49	156	53.1	100	46.9		77	28.1	183	71.9		46	26.0	133	74.0	
**Age at 1st birth**															
10–19	2147	41.1	1819	58.9	< 0.001	985	23.7	3013	76.3	< 0.001	543	20.3	2085	79.7	< 0.001
20–24	3182	38.1	3157	61.9		1488	23.7	4879	76.3		1041	24.3	3337	75.7	
25–29	1679	48.2	1323	51.8		942	33.3	2071	66.7		672	32.5	1419	67.5	
30–34	439	53.4	277	46.6		249	37.7	468	62.3		171	39.4	320	60.6	
35–49	110	58.5	64	41.5		55	33.9	119	66.1		47	42.1	67	57.9	
**Level of education**															
No education	114	69.4	27	30.6	< 0.001	64	40.8	84	59.2	< 0.001	20	29.3	63	70.7	< 0.001
Primary	1846	41	1588	59.0		926	25.6	2546	74.4		469	20.3	1828	79.7	
Secondary	3944	37.4	4136	62.6		1909	23.6	6196	76.4		1272	23.1	4251	76.9	
higher	1653	59.9	889	40.1		820	38.3	1724	61.7		713	44.3	713	55.7	
**Employment status**															
Not working	3422	39.8	3252	60.2	< 0.001	1687	25.6	5020	74.4	0.061	1044	23.5	3375	76.5	< 0.001
Working	4135	44.1	3388	55.9		2032	27.3	5530	72.7		1430	28.0	3853	72.0	
**Husband’s level of education**															
No education	130	62.8	38	37.2	< 0.001	73	39.1	105	60.9	< 0.001	23	21.7	94	78.3	< 0.001
Primary	1964	40.9	1701	59.1		947	24.6	2748	75.4		490	20.6	1922	79.4	
Secondary	4008	37.5	4193	62.5		2001	24.5	6232	75.5		1338	23.7	4294	76.3	
higher	1455	62.2	708	37.8		698	38.0	1465	62.0		623	45.4	918	54.6	
**Marital status**															
Married	7369	41.6	6626	58.4	< 0.001	3663	26.4	10,403	73.6	0.131	2454	25.9	7139	74.1	0.895
other	188	89.5	14	10.5		56	32.6	147	67.4		20	26.6	89	73.4	
**Parity**															
Primigravida	2130	37.8	2249	62.2	< 0.001	1141	26.8	3241	73.2	0.034	835	27.7	2228	72.3	0.022
Multigravida	4748	43.1	4071	56.9		2267	26.0	6598	74.0		1513	25.2	4507	74.8	
Grandemultigravida	679	55.8	320	44.2		311	31.1	711	68.9		126	21.9	493	78.1	
**Health insurance**															
No	2395	34	2884	66.0	< 0.001	1220	22.8	4101	77.2	< 0.001	735	20.8	2883	79.2	< 0.001
yes	5162	47.4	3756	52.6		2499	29.0	6449	71.0		1739	29.3	4345	70.7	
**Complication**															
Yes	1372	45.1	1119	54.9	0.0047	787	34.5	1708	65.5	< 0.001	559	34.2	1153	65.8	< 0.001
No	6185	41.3	5521	58.7		2932	24.7	8842	75.3		1915	24.1	6075	75.9	
**ANC Visit**															
no visits						48	59.9	29	40.1	< 0.001	18	46.5	17	53.5	0.116
< 4 times	645	44.6	512	55.4	0.1572	366	32.7	789	67.3		152	23.4	484	76.6	
≥ 4 times	6912	41.8	6128	58.2		3305	25.9	9732	74.1		2304	25.9	6727	74.1	
**Delivery type**															
Normal	N/A					2130	17.6	9604	82.4	< 0.001	1619	20.6	6338	79.4	< 0.001
Sectio						1589	66.7	946	33.3		855	49.1	890	50.9	
**Mass Media consumption**															
**News papers or magazines**															
No	4150	39.3	3878	60.7	< 0.001	2135	26.2	5945	73.8	0.433	1258	24.0	4118	76.0	< 0.001
Yes	3407	46.3	2762	53.7		1584	27.0	4605	73.0		1216	28.8	3110	71.2	
**Radio listening**															
No	4526	40.6	3963	59.4	0.002	2207	25.9	6335	74.1	0.160	1370	24.5	4350	75.5	0.003
Yes	3031	44.1	2677	55.9		1512	27.4	4215	72.6		1104	28.0	2878	72.0	
**Watching TV**															
No	447	59.1	164	40.9	< 0.001	220	34.7	403	65.3	0.002	81	26.6	251	73.4	0.851
Yes	7110	41.4	6476	58.6		3499	26.2	10,147	73.8		2393	25.9	6977	74.1	
**Wealth index**															
Lowest	2387	53.3	1129	46.7	< 0.001	1035	28.9	2530	71.1	< 0.001	439	20.6	1824	79.4	< 0.001
Second	1425	38.6	1401	61.4		583	20.6	2254	79.4		379	18.0	1556	82.0	
Middle	1180	34	1543	66.0		551	20.9	2180	79.1		406	20.4	1519	79.6	
Fourth	1172	34.8	1461	65.2		638	24.7	1998	75.3		475	25.4	1359	74.6	
Highest	1393	51	1106	49.0		912	38.2	1588	61.8		775	44.9	970	55.1	
**Type of residence**															
urban	3573	42.3	3531	57.7	0.676	1950	28.6	5172	71.4	< 0.001	1561	32.5	3269	67.5	< 0.001
Rural	3984	41.7	3109	58.3		1769	24.5	5378	75.5		913	19.7	3959	80.3	
**Province**															
Western Indonesia	3172	34.6	5436	65.4	< 0.001	2297	26.6	6356	73.4	0.037	1492	25.6	4335	74.4	< 0.001
Central Indonesia	3287	70.2	1062	29.8		1047	25.1	3314	74.9		777	25.4	2378	74.6	
Eastern Indonesia	1098	91.7	142	8.3		375	32.6	880	67.4		205	40.0	515	60.0	

Table 3 shows multiple logistic regression which analyze several significant determinants influencing the utilization of midwifery services for antenatal care (ANC), intranatal care (INC), and postnatal care (PNC) among Indonesian women in 2012.

For ANC, women who were married (AOR = 1.39; *p* = 0.031) and primigravida (AOR = 1.37; *p* = 0.034) showed significantly higher odds of receiving antenatal care from midwives. Those without health insurance were also more likely to use midwifery services from midwife (AOR = 1.32; *p* < 0.001). Wealth was positively associated with ANC utilization, with higher odds among women in the middle (AOR = 1.27; *p* = 0.014) and richer quintiles (AOR = 1.31; *p* = 0.003). Rural residence (AOR = 1.24; *p* = 0.006) was linked to greater midwife involvement. Additionally, regional disparities were notable, with women in the western (AOR = 13.10; *p* < 0.001) and central (AOR = 3.13; *p* < 0.001) provinces being significantly more likely to receive ANC from midwives compared to those in the eastern region.

For INC, significant predictors included age 35–39 (AOR = 1.68; *p* = 0.016) and first birth between 20 and 24 years (AOR = 2.15; *p* = 0.020). Maternal education showed a strong positive relationship, with women having primary (AOR = 2.00; *p* = 0.001), secondary (AOR = 2.97; *p* < 0.001), and higher education (AOR = 2.23; *p* = 0.001) being more likely to deliver with a midwife. Parity was also significant: primigravida (AOR = 1.75; *p* < 0.001) and multigravida women (AOR = 1.38; *p* = 0.005) had higher odds of receiving care. The number of ANC visits was a strong predictor: women with 1–3 visits (AOR = 2.37; *p* < 0.001) and ≥ 4 visits (AOR = 4.67; *p* < 0.001) were significantly more likely to receive INC from midwives. Normal delivery (AOR = 5.90; *p* < 0.001) was the strongest determinant. Higher wealth quintiles and regional location (western: AOR = 2.47; middle: AOR = 1.97; both *p* < 0.001) also increased the likelihood of midwife involvement.

For PNC, women who had their first birth at younger ages (10–19: AOR = 3.17; *p* = 0.003; 20–24: AOR = 3.67; *p* = 0.001; 25–29: AOR = 3.02; *p* = 0.003; 30–34: AOR = 2.36; *p* = 0.036) were more likely to receive postnatal care from midwives. Secondary education (AOR = 2.26; *p* = 0.006) was also positively associated. As with INC, a higher number of ANC visits significantly increased PNC use (1–3 visits: AOR = 3.01; *p* < 0.001; ≥4 visits: AOR = 3.82; *p* < 0.001). Women who had normal births deliveries (AOR = 8.64; *p* < 0.001) and those without complications (AOR = 1.29; *p* = 0.008) were more likely to receive midwifery care. Higher wealth status (poor to richer: AORs 1.61–1.67; all *p* < 0.001), rural residence (AOR = 1.27; *p* = 0.007), and location in the western (AOR = 1.96; *p* < 0.001) or middle (AOR = 1.60; *p* = 0.002) provinces were also significant predictors. Overall, utilization of maternal care from midwives in 2012 was strongly influenced by sociodemographic, obstetric, and regional factors, with clear disparities based on wealth, geographic location, parity, and prior healthcare-seeking behavior.

**Table 3:** Adjusted odds ratio and 95 confidence interval of ANC, INC, and PNC services from midwives over other health professionals by background characteristics: Indonesia DHS 2012

Characteristics		2012										
			ANC				INC				PNC	
	*p*-value	AOR	Lower	upper	*p*-value	AOR	Lower	upper	*p*-value	AOR	Lower	upper
**Age**												
15–19		1(ref)				1(ref)				1(ref)		
20–24	0.426	1.141	0.824	1.580	0.881	0.973	0.681	1.391	0.614	0.905	0.614	1.334
25–29	0.106	1.321	0.943	1.852	0.132	1.335	0.917	1.945	0.630	1.101	0.744	1.629
30–34	0.276	1.228	0.849	1.776	0.267	1.256	0.839	1.880	0.882	0.967	0.619	1.511
35–39	0.572	1.118	0.759	1.646	0.016	1.681	1.100	2.568	0.721	1.091	0.676	1.762
40–44	0.528	1.146	0.750	1.752	0.074	1.537	0.959	2.461	0.804	1.072	0.618	1.862
45–49	0.276	1.410	0.759	2.617	0.112	1.607	0.896	2.883	0.473	1.334	0.607	2.930
**Age at 1st birth**												
10–19	0.223	1.430	0.805	2.539	0.054	1.890	0.988	3.614	0.003	3.172	1.463	6.877
20–24	0.214	1.422	0.816	2.477	0.020	2.153	1.130	4.102	0.001	3.673	1.726	7.818
25–29	0.501	1.213	0.692	2.126	0.055	1.846	0.986	3.458	0.003	3.022	1.443	6.330
30–34	0.598	1.152	0.681	1.950	0.678	1.157	0.580	2.307	0.036	2.355	1.060	5.231
35–49		1(ref)					1(ref)			1(ref)		
**Level of education**												
No education		1(ref)				1(ref)				1(ref)		
Primary	0.399	1.228	0.762	1.977	0.001	2.005	1.330	3.022	0.065	1.754	0.966	3.185
Secondary	0.183	1.408	0.851	2.328	< 0.001	2.974	1.957	4.520	0.006	2.260	1.263	4.043
Higher	0.246	0.721	0.415	1.253	0.001	2.237	1.397	3.583	0.330	1.371	0.727	2.587
**Employment status**												
Not working		1(ref)				1(ref)				1(ref)		
Working	0.706	0.978	0.874	1.096	0.058	0.888	0.786	1.004	0.170	0.896	0.766	1.048
**Husband’s level of education**												
No education		1(ref)				1(ref)				1(ref)		
Primary	0.180	0.771	0.527	1.128	0.701	1.108	0.656	1.873	0.863	1.063	0.531	2.130
Secondary	0.162	0.758	0.514	1.118	0.111	1.539	0.905	2.617	0.759	1.116	0.554	2.250
higher	0.001	0.457	0.291	0.717	0.889	1.042	0.585	1.856	0.200	0.622	0.301	1.287
**Marital status**												
Married	0.031	1.395	1.030	1.889	0.820	0.963	0.693	1.336	0.900	1.027	0.679	1.554
other		1(ref)				1(ref)				1(ref)		
**Parity**												
Primigravida	0.034	1.369	1.024	1.830	< 0.001	1.756	1.299	2.373	0.184	1.286	0.887	1.866
Multigravida	0.923	1.012	0.791	1.295	0.005	1.384	1.103	1.737	0.392	1.141	0.844	1.543
Grandemultigravida		1(ref)				1(ref)				1(ref)		
**Health insurance**												
No	< 0.001	1.319	1.168	1.490	0.973	1.002	0.885	1.135	0.146	1.116	0.963	1.293
yes		1(ref)				1(ref)				1(ref)		
**Complication**												
Yes		1(ref)				1(ref)				1(ref)		
No	0.195	0.903	0.775	1.053	0.986	0.998	0.841	1.185	0.008	1.295	1.070	1.566
**ANC Visit**												
no visits						1(ref)				1(ref)		
1–3 times		1(ref)			< 0.001	2.372	1.725	3.263	< 0.001	3.006	1.749	5.168
>=4 times	0.895	1.012	0.845	1.213	< 0.001	4.675	3.513	6.222	< 0.001	3.818	2.317	6.291
**Birth Delivery type**												
Normal		NA			< 0.001	5.906	4.912	7.101	< 0.001	8.640	7.024	10.629
Sectio						1(ref)				1(ref)		
**Mass Media consumption**												
**News papers or magazines**												
No		1(ref)				1(ref)				1(ref)		
Yes	0.269	0.931	0.819	1.057	0.615	1.035	0.905	1.185	0.950	1.005	0.848	1.192
**Radio listening**												
No		1(ref)				1(ref)				1(ref)		
Yes	0.532	0.961	0.849	1.088	0.901	0.992	0.873	1.127	0.407	0.936	0.800	1.094
**Watching TV**												
No		1(ref)				1(ref)				1(ref)		
Yes	0.178	1.210	0.917	1.598	0.186	1.177	0.925	1.498	0.620	0.898	0.588	1.373
**Wealth index**												
Lowest	< 0.001	0.652	0.522	0.813	0.003	0.704	0.557	0.891	0.085	1.293	0.965	1.733
Second	0.665	0.958	0.788	1.165	0.103	1.188	0.966	1.461	< 0.001	1.615	1.240	2.104
Middle	0.014	1.272	1.051	1.539	< 0.001	1.560	1.276	1.907	< 0.001	1.839	1.417	2.387
Fourth	0.003	1.306	1.097	1.555	0.001	1.408	1.155	1.716	< 0.001	1.677	1.289	2.181
Highest		1(ref)				1(ref)				1(ref)		
**Type of residence**												
Urban		1(ref)				1(ref)				1(ref)		
Rural	0.006	1.248	1.065	1.463	0.744	0.973	0.825	1.147	0.007	1.274	1.068	1.518
**Province**												
Western Indonesia	< 0.001	13.102	9.066	18.933	< 0.001	2.471	1.970	3.099	< 0.001	1.962	1.463	2.631
Central Indonesia	< 0.001	3.125	2.146	4.552	< 0.001	1.967	1.560	2.480	0.002	1.602	1.197	2.143
Eastern Indonesia		1(ref)				1(ref)				1(ref)		

Table 4. presents multiple logistic regression which revealed significant determinants of maternal health service utilization by midwives for antenatal care (ANC), intranatal care (INC), and postnatal care (PNC) in 2017.

For ANC utilization, women who had their first birth at ages 10–19 (AOR = 1.81; *p* = 0.008) and 20–24 (AOR = 1.94; *p* = 0.002) were significantly more likely to receive care from midwives compared to those who had their first birth at ≥ 25 years. First-time mothers (primigravida) also showed higher odds of using midwife services (AOR = 1.55; *p* = 0.002). Lack of health insurance was associated with increased midwifery care utilization (AOR = 1.52; *p* < 0.001), suggesting accessibility or affordability of midwifery services from midwives among uninsured women. Additionally, women whose husbands had primary (AOR = 1.65; *p* = 0.037) or secondary education (AOR = 1.76; *p* = 0.021) were more likely to use ANC from midwives. Working women (AOR = 1.11; *p* = 0.038) and those from wealthier households (middle: AOR = 1.40; richer: AOR = 1.44; both *p* < 0.001) also showed significantly higher odds of midwifery service utilization. Regional disparities were evident, with markedly higher utilization in the western (AOR = 16.99; *p* < 0.001) and middle (AOR = 4.16; *p* < 0.001) regions compared to the east.

For INC, women with secondary education (AOR = 1.90; *p* = 0.010) were more likely to deliver with midwife assistance. Husband’s education also played a role, with primary (AOR = 1.55; *p* = 0.038) and secondary education (AOR = 1.76; *p* = 0.008) positively associated with midwife-assisted delivery. Women who experienced no complications during childbirth had higher odds of midwife-attended deliveries (AOR = 1.23; *p* = 0.003). The number of ANC visits was a strong predictor, with those attending 1–3 visits (AOR = 3.01; *p* = 0.002) or ≥ 4 visits (AOR = 5.54; *p* < 0.001) significantly more likely to receive INC from a midwife. Normal delivery was a dominant predictor (AOR = 10.19; *p* < 0.001), as was newspaper exposure (AOR = 1.15; *p* = 0.023). Women from higher wealth quintiles and those living in western and middle Indonesia also had greater odds of using midwife services during delivery.

For PNC, women aged 40–44 had significantly higher odds of receiving midwife care (AOR = 1.71; *p* = 0.046). Lack of health insurance (AOR = 1.21; *p* = 0.005) and absence of complications (AOR = 1.38; *p* < 0.001) were associated with increased utilization. Similar to INC, the number of ANC visits strongly influenced PNC use (1–3 visits: AOR = 3.66; *p* = 0.015; ≥4 visits: AOR = 4.35; *p* = 0.005). Normal birth delivery was again a strong predictor (AOR = 2.94; *p* < 0.001). Women in the richer wealth quintiles were more likely to use PNC services from midwives (AORs 1.84–2.19; *p* < 0.001). Rural residence was also associated with higher midwife service utilization (AOR = 1.48; *p* < 0.001), as were western (AOR = 2.82; *p* < 0.001) and middle (AOR = 2.64; *p* < 0.001) regions.

**Table 4:** Adjusted odds ratio and 95 confidence interval of ANC, INC, and PNC services from midwives over other health professionals by background characteristics: Indonesia DHS 2017

Characteristics		2017										
			ANC				INC				PNC	
	*p*-value	AOR	Lower	upper	*p*-value	AOR	Lower	upper	*p*-value	AOR	Lower	upper
**Age**												
15–19		1(ref)				1(ref)				1(ref)		
20–24	0.586	1.096	0.788	1.525	0.352	0.839	0.579	1.215	0.472	1.176	0.756	1.828
25–29	0.497	1.122	0.805	1.566	0.314	0.822	0.561	1.204	0.469	1.187	0.746	1.887
30–34	0.544	1.117	0.781	1.6	0.42	0.846	0.564	1.27	0.086	1.518	0.943	2.443
35–39	0.457	1.149	0.796	1.659	0.33	0.808	0.527	1.241	0.167	1.414	0.865	2.311
40–44	0.906	1.024	0.694	1.509	0.655	0.901	0.57	1.424	0.046	1.714	1.011	2.908
45–49	0.872	0.958	0.567	1.618	0.998	1.001	0.559	1.792	0.146	1.643	0.841	3.21
**Age at 1st birth**												
10–19	0.008	1.809	1.165	2.811	0.07	0.613	0.361	1.041	0.059	1.707	0.981	2.972
20–24	0.002	1.937	1.265	2.965	0.126	0.671	0.403	1.119	0.105	1.551	0.912	2.638
25–29	0.056	1.507	0.99	2.292	0.021	0.553	0.334	0.915	0.155	1.457	0.867	2.448
30–34	0.354	1.234	0.791	1.927	0.059	0.607	0.361	1.02	0.733	1.098	0.642	1.876
35–49		1(ref)				1(ref)				1(ref)		
**Level of education**												
No education		1(ref)				1(ref)				1(ref)		
Primary	0.071	1.835	0.95	3.545	0.078	1.539	0.953	2.487	0.252	1.512	0.745	3.066
Secondary	0.057	1.896	0.982	3.66	0.01	1.897	1.166	3.084	0.148	1.688	0.83	3.435
higher	0.455	1.291	0.66	2.525	0.092	1.563	0.93	2.627	0.616	1.206	0.581	2.504
**Employment status**												
Not working		1(ref)				1(ref)				1(ref)		
Working	0.038	1.109	1.006	1.222	0.262	1.067	0.953	1.194	0.468	0.952	0.834	1.087
**Husband’s level of education**												
No education		1(ref)				1(ref)				1(ref)		
Primary	0.037	1.654	1.032	2.651	0.038	1.547	1.024	2.335	0.725	0.888	0.458	1.721
Secondary	0.021	1.762	1.09	2.847	0.008	1.763	1.156	2.687	0.94	1.026	0.529	1.99
Higher	0.946	0.983	0.596	1.622	0.143	1.407	0.89	2.225	0.402	0.746	0.376	1.481
**Marital status**												
Married	< 0.001	3.628	1.865	7.058	0.711	1.089	0.695	1.706	0.573	0.853	0.491	1.482
other		1(ref)				1(ref)				1(ref)		
**Parity**												
Primigravida	0.002	1.553	1.182	2.041	0.391	1.138	0.847	1.528	0.903	0.977	0.676	1.413
Multigravida	0.113	1.202	0.957	1.508	0.086	1.213	0.973	1.513	0.792	0.959	0.705	1.306
Grandemultigravida		1(ref)				1(ref)				1(ref)		
**Health insurance**												
No	< 0.001	1.524	1.381	1.681	0.15	1.086	0.971	1.215	0.005	1.207	1.058	1.377
Yes		1(ref)				1(ref)				1(ref)		
**Complication**												
Yes		1(ref)				1(ref)				1(ref)		
No	0.013	1.166	1.034	1.315	0.003	1.231	1.073	1.412	< 0.001	1.378	1.193	1.592
**ANC Visit**												
no visits						1(ref)				1(ref)		
1–3 times		1(ref)			0.002	3.013	1.484	6.118	0.015	3.664	1.285	10.446
>=4 times	0.647	1.046	0.864	1.266	< 0.001	5.544	2.796	10.993	0.005	4.346	1.553	12.162
**Birth Delivery type**												
Normal		N/A			< 0.001	10.193	8.95	11.61	< 0.001	2.94	2.54	3.404
Sectio						1(ref)				1(ref)		
**Mass Media consumption**												
**News papers or magazines**												
No		1(ref)				1(ref)				1(ref)		
Yes	0.361	0.951	0.852	1.06	0.023	1.154	1.02	1.306	0.058	1.143	0.995	1.313
**Radio listening**												
No		1(ref)				1(ref)				1(ref)		
Yes	0.894	0.993	0.893	1.104	0.582	0.967	0.858	1.09	0.456	0.952	0.838	1.083
**Watching TV**												
No		1(ref)				1(ref)				1(ref)		
Yes	0.06	1.288	0.99	1.677	0.134	1.237	0.937	1.633	0.911	1.022	0.694	1.506
**Wealth index**												
Lowest	0.17	0.866	0.705	1.064	0.742	0.965	0.778	1.195	< 0.001	1.847	1.448	2.356
Second	0.045	1.199	1.004	1.432	< 0.001	1.613	1.338	1.944	< 0.001	2.189	1.769	2.708
Middle	< 0.001	1.403	1.181	1.666	< 0.001	1.701	1.419	2.038	< 0.001	2.074	1.699	2.531
Fourth	< 0.001	1.439	1.223	1.692	< 0.001	1.629	1.391	1.909	< 0.001	1.84	1.544	2.193
Highest		1(ref)				1(ref)				1(ref)		
**Type of residence**												
Urban		1(ref)				1(ref)				1(ref)		
Rural	0.115	1.109	0.975	1.26	0.893	0.991	0.863	1.137	< 0.001	1.477	1.267	1.721
**Province**												
Western Indonesia	< 0.001	16.987	12.82	22.508	0.03	1.366	1.031	1.809	< 0.001	2.824	2.084	3.827
Central Indonesia	< 0.001	4.157	3.096	5.582	0.004	1.525	1.147	2.029	< 0.001	2.643	1.93	3.619
Eastern Indonesia		1(ref)				1(ref)				1(ref)		

Note: AOR: Adjusted Odds Ratio; values represent the likelihood of utilizing midwifery services while controlling for other variables

## Discussion

This study highlights the pivotal role midwives play in providing maternal healthcare services in Indonesia. Across both 2012 and 2017 IDHS datasets, midwives consistently emerged as the primary providers of antenatal care (ANC), intranatal care (INC), and postnatal care (PNC), particularly among certain demographic groups. These findings highlight the importance of midwives in Indonesia’masternal health system, especially in community-based and accessible service delivery.

Younger women consistently demonstrated higher engagement with antenatal, intranatal, and postnatal care services from midwives compared to older age groups in both 2012 and 2017. Some programs that targeted young women, perceived approachability of village midwives within community, and strong family endorsement of *“ibu bidan”* in the community could be the outcomes of high utilization of midwife among younger population [35–37]. In 2022, early childbearers in 2012 were three times more likely to obtain PNC from midwives (AOR = 3.2–3.7) while women delivering their first child at 20–24 years were nearly twice as likely to receive ANC from a midwife (AOR = 1.94) in 2017. Younger women, especially adolescents and primiparous mothers, often view midwives as more approachable and easier to access compared to other healthcare professionals [38]. This perception is likely influenced by the fact that midwives frequently provide community-based services that are culturally sensitive and less formal, which may be highly appealing to younger women who are seeking maternal care [39]. Additionally, midwives hold a strong reputation in maternal healthcare across Indonesia. Familial and community norms may influence younger women’s utilization of midwifery services, which is consistent with cultural expectations and practices.

In terms of education, women with secondary education were almost three times more likely than the non-educated to give birth with a midwife in 2012 (AOR = 2.97) and twice as likely in 2017 (AOR = 1.90), underscoring the role of education in improving health-seeking behaviors. Multiple factors could account for this. Educational attainment can enhance an individual’s comprehension and awareness of health directives and health care [40, 41]. Higher levels of education are closely linked to improved health literacy, enabling women to make more informed choices about their healthcare providers. Women who are well-informed are more likely to seek out midwifery services, as they are aware of the benefits midwives offer, including lower rates of medical intervention and more personalized care. Prior research demonstrates women report greater satisfaction with their birth when care is provided by a midwife compared to a physician [42]. Another study also demonstrated that midwifery-led care during low-risk pregnancy and childbirth improved maternal outcomes and increased mothers’ satisfaction [43, 44].

Moreover, a husband’s level of education plays a notable role in shaping midwifery care utilization in 2012 and 2017. Previous sesearch indicated that higher educational attainment among husbands correlates positively with their involvement in antenatal care (ANC) visits and more likely to deliver at healthcare facilities [38, 45]. Educated husbands tend to better understand the risks associated with pregnancy and the importance of ANC [45]. Furthermore, husbands with higher levels of education are more likely to understand the benefits of professional healthcare and hold positive views toward maternal health services. This awareness often leads them to encourage their wives to seek care from qualified providers, such as midwives, who offer consistent support and individualized attention [46]. This awareness facilitates their involvement in health care decisions, which is essential given the patriarchal culture prevalent in Indonesia, where husbands often hold significant decision-making power regarding family health.

Employment status also played a complex role in maternal health care utilization. In 2012, unemployed women had enhanced access to midwifery services and utilized maternal health care (*n* = 59.6%), particularly from midwives. This trend may be attributed to their more flexible schedules, which allow them to attend antenatal visits more easily compared to employed women, who often face time constraints due to work commitments [46]. Moreover, physicians are considered healthcare professionals who specialize in the management of high-risk pregnancies and complex births who work in hospitals or private clinics which work mostly in urban areas [47]. Consequently, they are less frequently involved in the management of low-risk pregnancies and uncomplicated childbirth.

By 2017, however, the gap narrowed, and employed women showed improved odds of utilizing antenatal care (ANC) from midwives, with an AOR of 1.1 (95% CI: 1.0–1.2). To build on this progress, policies should focus on promoting workplace support for maternal health, such as paid maternity leave, on-site healthcare services, or partnerships with employers to encourage routine health checkups. Additionally, increasing awareness campaigns targeting both employed and non-employed women can ensure that all women, regardless of employment status, have equal opportunities to access essential maternal healthcare services.

Marital status was also a significant predictor in 2012. Married women were 1.39 times more likely to utilize ANC from midwives. Being married may increase access to care through spousal support and social acceptability, especially in conservative or rural communities where unmarried pregnancy is stigmatized. Midwives, as community-based providers, may be more accessible and acceptable to married women seeking discreet or locally available care.

Parity, or the number of children a woman has given birth to, is also a factor that can influence healthcare-seeking behavior. Mothers with higher parity are less likely to seek ANC and INC from midwives (ANC 2017 AOR = 1.55; INC 2012 AOR = 1.75). This could be because they feel more experienced and do not need as much care [48], especially if previous pregnancies were uneventful. A previous study found that mothers with five or more pregnancies were less likely to deliver with the help of a skilled birth attendant (SBA) (midwife, doctor, obstetrician, nurse) and did not make the recommended number of ANC visits [49]. Mothers with high parity status may be busy taking care of their children and household responsibilities, and may not have the time or resources to access ANC and INC services. However, further investigation is needed to fully understand. Given the elevated morbidity risk in grand multiparity [50, 51], midwives should intensify home-visit counselling on safe-birth planning and modern contraception [52]. For instance, midwives could offer counseling and access to a variety of contraceptive methods, as midwives hold key positions in the community through *Posyandu*,* Puskesmas*, and independent practices. Consequently, multigravida women may favor midwifery care for contraceptive services over the services of physicians and nurses.

In contrast, primigravida women utilized midwifery services from midwives more frequently than those nurses or physicians. Primiparous women frequently pursue accessible, community-oriented professional care, exemplified by maternal health services from midwife [46]. For the personalized support and guidance that midwives provide during pregnancy, childbirth, and postpartum, primiparous women may pursue maternal health care from midwife.

Uninsured women may be more likely to use midwifery services from midwives due to their affordability, accessibility, and integration into community-based health facilities like *Puskesmas*. Midwives offer low-cost care for uncomplicated pregnancies [53], making them a practical choice for women without health insurance. Their trusted presence in communities, combined with culturally sensitive and continuous care, reinforces their role as a preferred provider for uninsured women [54]. For example, midwives are well-trained to manage uncomplicated pregnancies and childbirth. Uninsured women, particularly those not experiencing complications, may prefer midwives for routine ANC, INC, and PNC due to trust in their services and a desire to avoid higher fees associated with specialist or hospital care.

Type of delivery also strongly influenced provider choice. Women who had normal deliveries were significantly more likely to use midwifery services from midwives. This confirms midwives’ key role in managing low-risk, physiological births (INC AOR = 10.19 in 2017; PNC AOR = 8.64 in 2012). As frontline providers, midwives are uniquely trained and trusted to deliver safe, evidence-based care during normal deliveries [55]. Their ability to offer personalized, culturally sensitive care further strengthens their position as the preferred choice for women experiencing uncomplicated births.

Another strong predictor of midwifery use was the number of ANC visits. Women who attended more ANC sessions were far more likely to continue care with midwives for delivery and postpartum services. This continuity builds trust, reinforces education, and ensures better outcomes [56]. Complication status was inversely associated with midwifery use for INC and PNC. Women without complications preferred midwives, while those with complications may have been referred to doctors or hospitals. This reflects appropriate task-shifting and highlights the midwife’s role as a frontline provider for low-risk cases [57].

Exposure to mass media—especially watching television and reading newspapers—was linked to greater use of midwifery services from midwives. This suggests that media can be a powerful tool for midwives to reach and educate the public. With their wide audience, television and print media can help midwives share important message, raise awareness about available services, and encourage women to start antenatal care early. By delivering information in ways that are culturally appropriate and easy to understand, midwives can help dispel myths and inspire women to seek timely care. Collaborating with media platforms also has the potential to build trust and strengthen the visibility of maternal health care as a vital part of community healthcare [58].

The study found women with higher wealth index were more engaged in midwifery services. Women with a higher wealth index were more likely to engage with midwifery services from midwives, possibly due to their greater ability to access preferred providers and demand more personalized care [46]. Midwives often offer a woman-centered approach that emphasizes continuity, respect, and support—qualities that may appeal to wealthier women seeking high-quality, less medicalized birth experiences. This aligns with previous findings showing that financially better-off women are more likely to seek care from providers they perceive as attentive and trustworthy, including midwives [59].

Higher wealth status led women to use midwifery services, perhaps because they had sufficient funds to pay for care. In contrast to other healthcare professionals, such as doctors or nurses, midwife provides personalized support throughout pregnancy, birth, and postpartum and also provides continuity of care. This method has been linked to improved outcomes and increased satisfaction, which may be appealing to women with higher socioeconomic status who are seeking personalized care. Women with lower economic status tend to experience cost as a barrier despite free ANC services. Access to healthcare facilities frequently necessitates dependable transportation, which may be inaccessible or unaffordable for economically disadvantaged women, irrespective of the healthcare providers they pursue [60]. Women facing pregnancy complications, living far from healthcare facilities, lacking health insurance, or residing in certain regions were found to encounter additional barriers [61].

Rural women were more likely to use ANC in 2012 and PNC in 2017 from midwives (ANC 2012 AOR = 1.24; PNC 2017 AOR = 1.48), reflecting the success of the village midwife programme. However, In 2017, western women were up to 17 times more likely than eastern women to receive ANC from a midwife (AOR = 16.99 in 2017). This disparity reflects the critical shortage of skilled birth attendants, including midwives, in areas such as Papua and Maluku [28]. The situation is further worsened by challenging geographical conditions and the concentration of healthcare facilities in densely populated central regions, making access difficult for rural communities [28].

In summary, multiple factors influenced midwifery service utilization in Indonesia between 2012 and 2017. Midwives remain central to maternal healthcare delivery, especially among young, educated, uninsured, and rural populations. Future programs should enhance continuity of care with midwives, improve insurance coverage and public awareness, and target disparities in access and outcomes. Strengthening the role of midwives within the healthcare system—particularly in underserved regions—will be critical to achieving maternal and neonatal health goals.

### Implications for future programs and research

This study underscores the need to strengthen and expand the use of midwifery services by midwives, especially in underserved areas. Future programs should ensure equitable access to midwife-led care across all groups, regardless of age, parity, education, employment, or location. Key priorities include improving continuity of care through community-based midwifery, addressing educational disparities via health literacy efforts, and expanding midwife availability in rural and eastern regions. Involving male partners, utilizing mass media to raise awareness, and promoting national health insurance benefits that cover midwifery services are also essential. Further research should explore women’s decision-making processes and assess the impact of midwife-led care, particularly in regions with low service coverage, to inform effective and scalable strategies.

### Strengths and limitations

This study has several important strengths. This study uses large, nationally representative data from the 2012 and 2017 Indonesia Demographic and Health Surveys (IDHS), which means the findings reflect a broad picture of maternal health service use across the country. By focusing specifically on care provided by midwives, the study highlights how essential their role is in supporting women throughout pregnancy, childbirth, and after birth. Moreover, multiple regression analysis helps identify which groups of women are more or less likely to use midwifery services, while comparing data across two years gives valuable insights over a period of time.

However, it is essential to acknowledge the limitations of this study. The cross-sectional study design, which collects data at a specific point in time, inherently restricts the ability to establish causative relationships between variables. Additionally, the data may not offer a complete representation of the entire Indonesian population. Moreover, as data collection occurs at distinct intervals, it may not always reflect the most up-to-date conditions. These limitations should be considered when interpreting the research findings, as they may impact the accuracy and informativeness of the results.

## Conclusion

This study highlights how vital midwives are to Indonesia’s maternal healthcare system, showing a clear increase in the use of midwifery services between 2012 and 2017. The results reveal that a woman’s age, education, work status, number of children, income level, and where she lives were influenced by whether she turned to a midwife for care. These factors shape her access to and experience with maternal health services.

Midwives continue to be the heart of maternal care, especially in rural and remote communities. They offer respectful, culturally sensitive, and accessible support throughout a woman’s journey—from pregnancy to childbirth and beyond. To improve maternal and newborn health, Indonesia must keep investing in midwifery. Making midwife-led care available to all women, especially in underserved areas, is not just a health policy goal; it is a key part of ensuring fairness, dignity, and quality care for every mother in the country.

## Data Availability

The dataset analyzed during the current study is available in the Demographic and Health Surveys (DHS) at https://dhsprogram.com/Data/.

## Abbreviations

ANC: Antenatal care
INC: Intranatal care
PNC: Postnatal care
SBA: Skilled Birth Attendant(midwives and doctors)
SDGs: Sustainable Development Goals
WHO: World Health Organization
